# Uterine artery embolization for leioyomas, ultrasonography and angiography aspects

**Published:** 2012-12-25

**Authors:** IA Horhoianu, VV Horhoianu, D Joita, M Carstoiu, B Dorobat

**Affiliations:** * "Carol Davila" University of Medicine and Pharmacy, Bucharest; ** Gynecology Department University Emergency Hospital, Bucharest; *** "Titu Mairescu" University, Informatics Department

**Keywords:** anastomoses, volume, decrease, fibroid, vascularization

## Abstract

**Objective and Rationale.** The purpose of this study is to evaluate the degree of fibroid shrinkage which well correlates with symptom regression, and thus to assess the effectiveness of the procedure

**Method.** 31 patients were included in the trial after selection. All the patients were thoroughly evaluated before embolization, the following day and at a month after but also at 3 months for 10 of them. A certain protocol was followed passing through well established steps. The purpose was to discover and rule out any associated possible disease and to assess and grade the symptoms, ultrasound and angiographic aspects.

**Results.** Regarding the uterus, its volume evolution registered a descending trend, the mean decrease at 30 days being of 25% (-81,6 cm³) and at 90 days of 52%(-173,15 cm³). The fibroids also decreased statistically, the mean global variation at 30 days registering a decrease of -17,66 cm³(27%) and 61% at day 90. The mean global reduction at 30 days was of 44% (-33,18 cm³) and of 62% (-60,85 cm³) at 90 days. Absence of uterine anastomoses lead to proper fibroid decrease whereas their presence diminished the chances.

**Conclusions.** The uterine and fibroid volumes registered a statistical volume decrease at 30 and 90 days in comparison with the volumes before embolization. Absence of uterine anastomoses led to proper fibroid decrease. Longer evaluation time is needed for an accurate evaluation of volume reduction degree.

## Introduction

Leiomyomas are the most frequent benign uterine tumors encountered in gynecology at reproductive age [**1**]. Though going in most cases unnoticed, many patients discover having fibroids after complaining about one of the following: bulk related symptoms, pain, infertility and bleeding related symptoms [**2,1**]. In conclusion, uterine fibroids are a modern day problem, which needs proper conservative and efficient treatment. There is continuous research regarding the development of an ideal method of minimally-invasiveness, safety and well tolerance for treating such cases. Many conservative operatory or non-operatory techniques have been developed: laparoscopic uterine artery ligation, myomectomy, high intensity focused ultrasound etc. among which uterine artery embolization is also cited [**1,3-5**]. According to Dr. Jaques Merland, the first uterine artery embolization procedure was made in 1974 on a patient with uncontrollable bleeding and poor candidate for operation; it was a success [**6**]. The first published report in English literature though appeared in 1995 by Dr Charles Ravina [**6-8**]. Our clinic in collaboration with the interventional radiology department has a large experience related to this field. In regards to this we consider uterine artery embolization to be a known, reproducible and safe procedure, not only used for the treatment of fibroids but also for uncontrollable hemorrhages (postpartum [**9,10**], arterial venous malformations [**11**], cervical end stage cancer [**12**], post curettage molar pregnancy [**13**]), ectopic pregnancy [**14**] and adenomyosis [**15**]. This technique obtains a fibroid reduction by obstructing its vessels with specially selected embolic particles via catheterization of the uterine arteries [**4**]. It is stated that leiomyoma shrinkage and devascularization is well correlated with symptom reduction and quality of life improvement [**5,16**].The purpose of this study is to evaluate the degree of shrinkage closely tied to clinical alleviation, and thus to assess the effectiveness of the cited method [**5,16**]. 

## Materials and methods

The study regarding uterine artery embolization of leiomyomas was held between 04.2011-05.2012 at the Bucharest University Emergency Hospital with approval of the institutional management and the informed consent of each patient. 60 patients were initially included in the trial, no sex or ethnic based differences being present; among those only 31 patients came to the follow-up thus remaining in the trial. All the patients were thoroughly evaluated before, the following day and at a month after embolization, 10 being also at 3 months assessed. A certain protocol was followed passing through well-established steps for each period. 

 Pre embolization protocol - All patients went through the following examinations: clinical evaluation; blood tests (full blood count, biochemistry, coagulogram, oxidative stress factors, hormones, growth factors); urine exam and urinary culture; pap smear, cervical and vaginal cultures; transvaginal ultrasound with Doppler examination. The purpose was to rule out any associated possible malignant disease, pregnancy or genito-urinary infections; to discover any other illnesses; and to assess and grade the symptoms, the location, type, number, complications, vascularization and volume of the uterus and fibroids. After a complete diagnosis, each patient was informed, a discussion taking place. The patients were ruled out or accepted based on the uterine artery fibroid embolization and angiography indications. The embolization procedure, evolution, possible risks and complications were explained, all patients giving an informed consent.

 Embolization procedure- In all patients the left brachial artery was catheterized up to the uterine arteries; upon failure, access via the right brachial artery was established. An angiogram showing the vascularization of the uterus, fibroma and communications (left to right and utero-ovarian anastomoses) was obtained. The next step consisted of bilateral occlusion of the vessels nourishing the fibroids by injecting 1 to 4 units of microspheres ranging from 355-1100 micrometers in diameter combined with half a unit gelaspon particles depending on the dimensions and vascularization of the arteries needing to be occluded. The angiographic end-point was obtained when stasis on the fibroid arteries occurred in combination with persistence of anterograde flow in the uterine arteries- the pruned tree angiographic appearance [**17**].

 Post-embolization hospital stay-The patient remained at bed rest until being able to resume normal functions. Immobilization of the former catheterized arm was considered necessary for 6 hours. Each patient was carefully monitored (blood pressure, oxygen saturation, diuresis, temperature, pain scale, symptoms) being medicated upon need for pain, and nausea. The second day another assessment took place: clinical examination and transvaginal ultrasound. The vast majority of patients had only an over-night stay, being let out the following for improvement of symptoms. Post-embolization protocol- The patients went through the same procedures as before embolization except the pap-smear at the following periods of time: 1 month, 3 months.

## Results

**Fig. 1:**
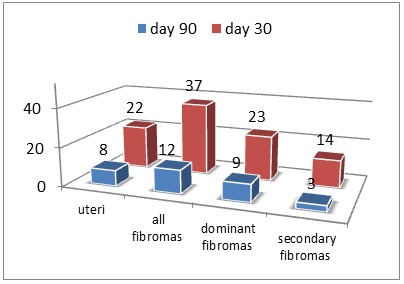
Number- Uterine and fibroma decrease

**Fig. 2:**
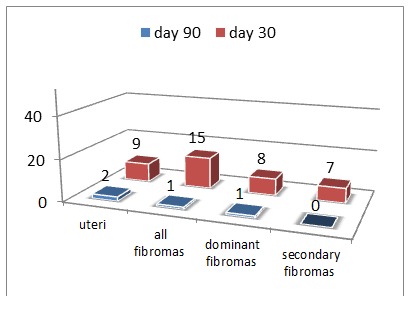
Number- Uterine and fibroma increase

Uterus and fibroid number, placement and trend – Out of the 31 patients evaluated at 30 days, 71% (22 patients) registered uterine volume reduction; only 10 came at de 90 days follow-up of which 80%(8 patients) registered a shrinkage (**Table 1**, **Fig. 1**, **2**). 

**Table 1:** Increase and decrease of uterus and fibromas

		uterus	All fibroids	Dominant fibrids	Secondary fibroids
Day 30	total	31 (100%)	52 (100%)	31 (60% )	21 (40%)
	shrinkage	22 ( 71%)	37 ( 71%)	23 (74%)	14 (66%)
	growth	9 ( 29%)	15 ( 29%)	8 (26%)	7 (33%)
Day 90	total	10 (100%)	13 (100%)	10 (77% )	3 (23%)
	shrinkage	8 (80%)	12 ( 92%)	9 (75%)	3 (25%)
	growth	2 (20%)	1 (8%)	1 (100%)	0 (0%)

 The total count of leiomyomas was of 52 out of which 60% (31 fibroids) were dominant, and thus of primary interest. The mean leiomyoma count was of 1,67±0,9 (range 1-4) with 18 patients having 1 fibroid, each 6 patients having 2 and 3 and only one having 4 fibroids. 71% (37) of fibroids registered a decrease at day 30 among which 23 (74%) were dominant; on day 90 of the 13 fibromas examined 77% (10) were dominant and 9 of them registered a decrease (75%) (Table 1, Fig. 1, 2). The 52 fibroids were divided according to uterine layers in: intramural 41 (79%), subserous 10 (19%) and submucous 1 (2%). 

**Fig. 3:**
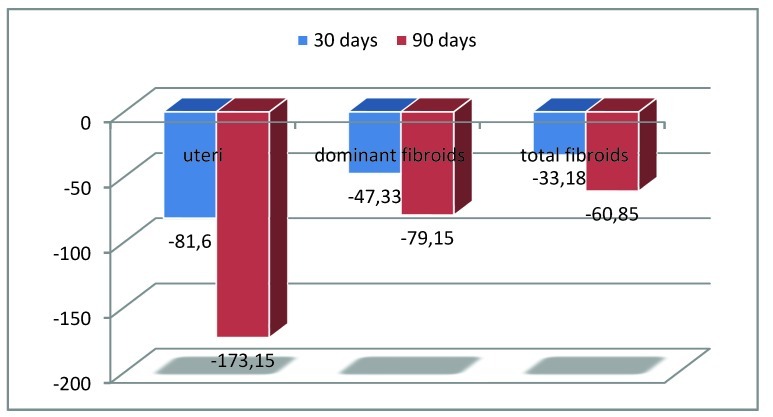
Volume- Uterine and fibroma decrease

 Uterine variation, shrinkage and growth –Variation- The mean uterine volume for the 0-30 day difference registered a variation from 293,60± 192,13 cm³ (range 712,82-81,63 cm³) to 248,98± 157,20 cm³ (range 606,01-70,93 cm³) reaching the conclusion that there is a global decreasing trend (Wilcoxon test, p=0,01) ; the 0-90 day difference varied between 296,63± 217,52 cm³ (range 712,82-106,5 cm³) and 173,20± 88,44 cm³ (range 299,17-77,99 cm³). Shrinkage-The mean uterine volume for the 0-30 days difference varied between 321,08± 205,75 cm³ before embolization (range 712,82-81,63cm³ ) and 239,47± 158,33 cm³ (range 600,44-70,93 cm³) 30 days after. For the 0-90 day difference the shrinkage ranged from 332,06±230,20 cm³ (extremes 712,82-106,88 cm³) to 158,91± 86,81 cm³ (range 297,06-77,99 cm³) (**Fig. 3**). 

In conclusion the mean decrease at 30 days was of 25% (-81,6 cm³) and at 90 days of 52% (-173,15 cm³). -Growth- For the 0-30 day difference the mean volume increased from 262,42±141,95 cm³ (range 512,83-106,5 cm³) to 174,18± 92,14 cm³ (range 606,01-123,1 cm³); for the 0-90 days difference respectively from 154,92± 68,47 cm³ (range 106,50-203,34 cm³) to 230,36± 97,32 cm³ (range 299,17-161,54 cm³). In conclusion, the mean growth at 30 days was of 20% (+45,78 cm³) and at 90 days of 49% (+75,44 cm³).

Fibroid variation, shrinkage and growth - General variation-Each fibroid has been separately analyzed, concluding to a general decreasing trend: the mean volume at day 0 for all fibroids was 64,27±77,88 cm³ (range 316,70 - 0,92 cm³) and reached 46,62 ± 55,87 cm³ (range 249,24-0,31 cm³) at 30 days, registering a descending trend ( p<0,001 Shapiro-Wilk test; Wilcoxon test p=0,0113; Kendall test p=0,0125 W=0,11982). For the 0-90 day difference the mean volume pre-embolization was 92,05± 89,72 cm³ (range 97,79-1,8 cm³4) and descended to 36,20± 35,98 cm³ (range 99,42-0 cm³) at day 90 (Wilcoxon test p=0,0028; Kendall test W=0.6944, p=0.00389). The mean variation at 30 days was of -17,66 cm³, -27% (dominant fibroids -29%, -27,63 cm³) and of -61% at day 90. (values -71,15 cm³ for dominant fibroids in comparison with -55,85 cm³ for all fibroids). -30 days evaluation- A global mean volume reduction occurred from day 0 76,22±85,30 cm³ (range 1,84-316,70 cm³) to 43,04±50 cm³ ( range 0,31-185,76 cm³) at day 30 for all the fibroids (dominant fibroids – day 0 110,35±90,6 cm³ ; day 30 63,02±53,72 cm³). The mean shrinkage was of 43% (-47,33 cm³) for the dominant fibroids and of 44% (-33,18 cm³) for the global number (**Fig. 3**). At 30 days the volume increased from 53,57±56,11 cm³ at day 0 to 82,57±85,97 cm³ at day 30 for the dominant fibroids (all fibroids –increased from 34,79±45,59 cm³ to 55,43±69,47 cm³) -90 days evaluation- For the 12 fibroids that decreased , the mean reduction was of 62% for both the dominant (-79,15 cm³ ) and total number of fibroids(-60,85 cm³ ) (**Fig. 3**). Only 1 out of the 13 leiomyomas grew in comparison to the pre-embolization volume, being a dominant one. The mean elevation was of 5% (0,88 cm³) in comparison with day 0, but it registered a downward trend in comparison with day 30 possibly continuing after 3 months with its descent; in conclusion longer time is needed for proper evaluation.

Utero-ovarian anastomoses

**Fig. 4:**
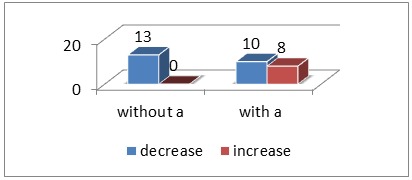
Volume- Uterine and fibroma decrease

We found such connections to be present in 58% of cases, 18 out of 31 patients. These were found before and or after particle injection stating a reflux from the uterine to the ovarian circulation. We decided to classify them into 3 types: indirect, direct, (the first communicating with the ovarian artery through ovarian anastomoses and the latter having a direct connection to the ovarian artery, appearing before particle injection) and reflux anastomoses (appearing after particle injection at the embolization endpoint). Unfortunately, lack of anastomoses does not necessarily mean their inexistence, possible vasospasm being a potential explanation for their hiding. After statistical analysis we concluded that a lack of such anastomoses led to fibroid reduction, whereas their presence assured growth in 56% of cases an shrinkage in 44%.(chi-square test p=0,009) (**Fig. 4**).

## Discussion

Regarding the uterine volume we concluded that the mean decrease at 30 days was of 25% (-81,60 cm³) and at 90 days of 52% (-173,15 cm³); similar results were found in literature: according to [**18**] the decrease was of 42% at 3 months (embolization with Embozene) and at 1 year up to 55% but with possible growth afterwards [**19**]. The fibroid volume decrease from our study appears to resemble similar studies in foreign literature; we concluded that the overall main fibroid volume registered a 44% reduction, -33,18 cm³ (43%, -47,33 cm³ for dominant fibroids) at 1 month and 62%, -60,58 (62%, -79,15 cm³ for dominant fibroids) at 3 months. In comparison [**17**] there was a 52% volume decrease at 3 months follow-up (p<0,001), the preinteventional mean volume of the dominant fibroid being 137,2+245,1 ml; another study, [**20**], registered a reduction of 52,62%±21,85% from the mean volume of 51,6 cm³ [**20**] . At one year [**20**] a 20,5%±11,92% decrease in comparison with the 3 months evaluation was added, 6%(5 tumors) being undetectable at that time and 8,3% (7 tumors) increasing in size. According to [**20**], 98,8% (83 tumors) experienced volume reduction at 3 months and at 1 year 85,7% (72 tumors) registered further decrease. 

 Regarding uterine anastomoses a number of 40,3% was found according to [**21**], 116/288 patients whereas in our study we described a percentage of 58%, 18/31 patients. According to the same study [**21**] a higher rate of repeat interventions was found among patients with uterine anastomoses at a mean period of 215 months (5 hysterectomies, 4 myomectomies, 5 repeat embolizations, meaning 12,1% (14 patients) of the patients with anastomoses, in comparison with 1,2% for the patients without any anastomoses (2 hysterectomies). In regards to location [4,5,20] stated that submucous fibroids experience the greatest reduction, the subserous ones registering the least ( p=0,26 at 3 months and p=0,0046 at 1 year for % in volume decrease). The mean decrease in stalk diameter for pedunculated fibroids appears to be of 0,3 cm (confidence interval 95%: 0,18-0,52 cm) or 13% from the initial diameter [**22**]. According to [**22**] the mean infarction rate for these fibroids is of 87% in comparison to a global of 92%, this leading to a more frequent inclusion in the poor clinical evolution group with smaller volume reduction (4 months evaluation) [**5**].There was no statistic significance on comparing fibroid location with volume decrease( p>0,05) in our study.

## Conclusion

Regarding the uterus, its volume evolution registered a descending trend, the mean decrease at 30 days being of 25% (-81,6 cm³) and at 90 days of 52% (-173,15 cm³). The fibroids also registered a statistical reduction, the mean global variation at 30 days being of -17,66 cm³ (27%) and of 61% (-55,85 cm³) at day 90. The mean shrinkage at 30 days was of 43% (-47,33 cm³) for the dominant fibroids and of 44% (-33,18 cm³) for the global number whereas at 90 days of 62% (-79,15 cm³ dominant fibroids; -60,58 cm³ total number of fibroids) for either group. After observing a continuous descending trend even for fibroids that initially grew, it was decided that longer time than 3 months was to be needed for proper evaluation. Absence of uterine anastomoses leads to reliable fibroid decrease whereas their presence diminishes the chances.
